# Association between thermogenic brown fat and genes under positive natural selection in circumpolar populations

**DOI:** 10.1186/s40101-024-00368-1

**Published:** 2024-08-19

**Authors:** Yuka Ishida, Mami Matsushita, Takeshi Yoneshiro, Masayuki Saito, Kazuhiro Nakayama

**Affiliations:** 1 Department of Integrated Biosciences, Graduate School of Frontier Sciences, The University of Tokyo, 5-1-5 Kashiwanoha, Kashiwa, Chiba 277-8562 Japan; 2 Department of Nutrition, School of Nursing and Nutrition, Tenshi College, Sapporo, Hokkaido 065-0013 Japan; 3 Division of Molecular Physiology and Metabolism, Tohoku University Graduate School of Medicine, Sendai, Miyagi 980-8575 Japan; 4 Laboratory of Biochemistry, Faculty of Veterinary Medicine, Hokkaido University, Sapporo, Hokkaido 060-0818 Japan

**Keywords:** Cold adaptation, Brown adipose tissue, Single nucleotide polymorphism, Metabolism, Positive natural selection

## Abstract

**Background:**

Adaptation to cold was essential for human migration across Eurasia. Non-shivering thermogenesis through brown adipose tissue (BAT) participates in cold adaptation because some genes involved in the differentiation and function of BAT exhibit signatures of positive natural selection in populations at high latitudes. Whether these genes are associated with the inter-individual variability in BAT thermogenesis remains unclear. In this study, we evaluated the potential associations between BAT activity and single nucleotide polymorphisms (SNPs) in candidate gene regions in East Asian populations.

**Methods:**

BAT activity induced by mild cold exposure was measured in 399 healthy Japanese men and women using fluorodeoxyglucose-positron emission tomography and computed tomography (FDG-PET/CT). The capacity for cold-induced thermogenesis and fat oxidation was measured in 56 men. Association analyses with physiological traits were performed for 11 SNPs at six loci (*LEPR*, *ANGPTL8*, *PLA2G2A*, *PLIN1*, *TBX15*-*WARS2*, and *FADS1*) reported to be under positive natural selection. Associations found in the FDG-PET/CT population were further validated in 84 healthy East Asian men and women, in whom BAT activity was measured using infrared thermography. Associations between the SNP genotypes and BAT activity or other related traits were tested using multiple logistic and linear regression models.

**Results:**

Of the 11 putative adaptive alleles of the six genes, two intronic SNPs in *LEPR* (rs1022981 and rs12405556) tended to be associated with higher BAT activity. However, these did not survive multiple test comparisons. Associations with lower body fat percentage, plasma triglyceride, insulin, and HOMA-IR levels were observed in the FDG-PET/CT population (*P* < 0.05). Other loci, including *TBX15-WARS2*, which is speculated to mediate cold adaptation in Greenland Inuits, did not show significant differences in BAT thermogenesis.

**Conclusions:**

Our results suggest a marginal but significant association between *LEPR* SNPs. However, robust supporting evidence was not established for the involvement of other loci under positive natural selection in cold adaptation through BAT thermogenesis in East Asian adults. Given the pleiotropic function of these genes, factors other than cold adaptation through BAT thermogenesis, such as diet adaptation, may contribute to positive natural selection at these loci.

**Supplementary Information:**

The online version contains supplementary material available at 10.1186/s40101-024-00368-1.

## Background

Homeothermic mammals need to maintain their body temperature at a nearly constant level to ensure proper functioning of their vital organs. Most mammals have developed strategies to maintain stable body temperatures, such as shivering or non-shivering thermogenesis and insulation provided by fur and subcutaneous fat [1]. Natural selection plays an important role in the evolution of these traits in mammalian species living in cold environments. Modern humans have long been believed to have evolved cold-adaptive phenotypes during their migration from Africa during the last glacial period. Compared to other mammals, humans have less body hair, a trait that is expected to make a limited contribution to insulation. The high capability for adaptive thermogenesis observed in human populations in cold climates, such as the Greenland Inuit, is regarded as a phenotype for coping with cold environments [2]. Non-shivering thermogenesis is primarily mediated by the brown adipose tissue (BAT). In cold environments, shivering thermogenesis produces heat and increases energy expenditure; however, after prolonged exposure to cold, shivering decreases gradually, and non-shivering thermogenesis mechanisms take over [3]. In humans, high BAT activity during cold exposure has been associated with reduced reliance on shivering and higher rectal temperatures [4]. Thus, non-shivering thermogenesis may play a more significant role in cold adaptation than shivering thermogenesis.

The general understanding regarding the genetic basis of cold adaptation via non-shivering thermogenesis remains fragmented, and only a few candidate loci have been reported to date. The gene encoding the mitochondrial uncoupling protein 1 (*UCP1*), which acts as a part of the thermogenic machinery in BAT, is involved in cold adaptation in modern humans. Single nucleotide polymorphisms (SNP) in *UCP1* have been linked to adaptive thermogenesis and the development of obesity [5–7]. This SNP has a specific geographical distribution, with a putative adaptive allele involved in high BAT thermogenesis and obesity resistance that is more frequent in high-latitude populations [6, 8]. SNPs in the adrenoceptor beta 2 and 3 genes are significantly associated with cold-induced activity in BAT [5, 9], although no firm evidence is available to reject their selective neutrality.

Several other candidate genes for cold adaptation in humans have been reported, mainly from population genetic studies [10–13]. Greenland Inuits have a genomic signature of natural selection related to adaptation to extreme conditions: a locus containing the T-box transcription factor 15 (*TBX15*) and tryptophanyl tRNA synthetase 2 (*WARS2*) genes and a cluster of genes encoding fatty acid desaturases (*FADS1–3*) [14–16]. In indigenous Siberian and Central and East Asian (EAs) living in cold climates, a specific variant that may be under positive natural selection was found in the phospholipase A2 group 2A gene (*PLA2G2A*). Angiopoietin-like-8 (*ANGPTL8*) and perilipin1 (*PLIN1*) also exhibit signatures of positive selection in these populations [17]. The allele frequencies of SNPs in the leptin receptor (*LEPR*), especially rs1137100 (Lys109Arg), are correlated with principal components representing winter in populations worldwide [11, 18]. Moreover, haplotypes consisting of rs1137100 and tightly linked SNPs exhibit increased homozygosity in EAs, implying recent positive selection at this locus [19, 20]. Although the products of these genes have been identified as molecules that participate in BAT differentiation and function in rodents [12, 17, 21, 22], it remains unclear whether these genes play a similar role in humans and whether the adaptive allele indeed renders cold tolerance in humans. We recently conducted an association study on BAT activity and successfully identified an SNP in the adrenoceptor beta 2 gene significantly associated with variability in cold-induced thermogenesis [9]. The set of genomic DNA and phenotypic data used in this study can help test putative cold-adaptive genes in humans. In this study, we evaluated the association between putative adaptive alleles of these genes and BAT activity to determine whether these polymorphisms are associated with cold adaptation in humans. In this study, we focused on EAs because the SNPs of interest are polymorphic in this population. In addition, the close genetic relationship with Arctic people and variability in their capability for adaptive thermogenesis [23] suggest that testing the association between these genes and cold adaptation in EAs is an appropriate strategy.

## Methods

### Study participants

BAT activity was assessed in two independent populations using two measurement methods, fluorodeoxyglucose-positron emission tomography and computed tomography (FDG-PET/CT) or infrared thermography (IRT), respectively. Details of the participants and the methods for measuring BAT activity have been described previously [5, 9, 24]. The FDG-PET/CT population included 399 healthy Japanese men and women (age, 27.1 ± 7.6; body mass index [BMI; kg/m^2^], 21.9 ± 2.9; Tables 1 and S1) who were living in or near Sapporo City, Hokkaido, Japan. The IRT population comprised 84 healthy men and women (age, 26.7 ± 7.0; BMI [kg/m^2^], 21.5 ± 3.0; Tables 2 and S2) who lived in or near Kashiwa City, Chiba, Japan, and included individuals of Japanese, Chinese, Korean, and Taiwanese origin. Their ethnic origins were Japanese (46), Chinese (35), Korean (2), and Taiwanese (1) (Table S3). The study design complied with the principles of the Declaration of Helsinki and was approved by the Ethics Committees of the authors’ institutions. All participants provided written informed consent prior to inclusion in these studies.

**Table 1:** Characteristics of participants in the FDG-PET/CT population

	All	BAT-positive	BAT-negative	*P*-value
*n* (men/women)	399 (343/56)	247 (225/22)	152 (118/34)	< 0.001
Age (years)	27.1 ± 7.6	25.6 ± 6.3	29.6 ± 9.0	< 0.001
Height (cm)	170.5 ± 7.3	171.0 ± 7.2	169.6 ± 7.3	0.759^b^
Weight (kg)	63.7 ± 10.5	63.0 ± 9.5	64.8 ± 11.8	0.006^b^
BMI (kg/m^2^)	21.9 ± 2.9	21.5 ± 2.6	22.5 ± 3.2	0.003^b^
BFP (%)	19.0 ± 6.4	17.8 ± 5.5	20.8 ± 7.1	0.189^b^
SUV_max_^a^	6.1 ± 6.3	7.9 ± 6.4	0.9 ± 0.4	< 0.001

*P*-values between BAT-positive and BAT-negative phenotypes were calculated as follows: men-to-women ratio, Pearson’s chi-square test; age and SUV_max_, Mann–Whitney *U* test; other traits, multiple linear regression model adjusted for age and sex. Continuous variables are presented as means ± standard deviation

^a^Data from 325 participants is shown (BAT-positive: *n* = 242; BAT-negative: *n* = 83)

^b^*P*-value shows the effect of BAT-positive or BAT-negative groups

*BAT* Brown adipose tissue, *BFP* Body fat percentage, *BMI* Body mass index, *FDG-PET/CT* Fluorodeoxyglucose-positron emission tomography and computed tomography, *SUV*_*max*_ Maximum standardized uptake value

**Table 2:** Body characteristics of the participants in the IRT population

	All (*n* = 84)
Age (years)	26.7 ± 7.0
Height (cm)	168.1 ± 8.4
Weight (kg)	60.7 ± 9.8
BMI (kg/m^2^)	21.5 ± 3.0
BFP (%)	23.9 ± 8.0
∆Temp (°C)	1.9 ± 0.9

Continuous variables are presented as means ± standard deviation

*BFP* Body fat percentage, *BMI* Body mass index, *ΔTemp* BAT activity calculated by subtracting the skin surface temperature in the control chest region from that in the supraclavicular fossa region, *IRT* Infrared thermography

### BAT measurements using FDG-PET/CT

BAT activity was measured during the winter season (from December to March) using a methodology established and utilized in previous studies. The participants wore light clothes (a T-shirt and underwear or a disposable lightweight gown) and stayed in a 19 °C room for 1 h after fasting for 6 to 12 h. Subsequently, they were administered ^18^F-fluoro-2-deoxyglucose (1.66–5.18 MBq/kg body weight) intravenously and exposed to the same cold condition for another hour. FDG-PET/CT examinations using a PET/CT system (Aquiduo; Toshiba Medical Systems, Ohtawara, Japan) were performed in a room at 24 °C. BAT activity in a region of interest was quantified by calculating the maximum standardized uptake value (SUV_max_). An SUV_max_ of 2.0 or greater was considered to indicate high BAT activity (BAT-positive), while an SUV_max_ of less than 2.0 indicated low activity (BAT-negative) [24, 25]. In 74 samples, BAT-positive/BAT-negative status was duly documented, but the raw SUV_max_ value was absent.

### Measurements of cold-induced thermogenesis (CIT) and fat oxidation (FO)

CIT and FO were measured in a 56-men subset of the FDG-PET/CT population in our previous studies [26, 27]. Whole-body energy expenditure was measured using a respiratory gas analyzer connected to a ventilated hood (AR-1; Arco System, Kashiwa, Japan). Energy expenditure adjusted for free fat mass (kg) was used to calculate the CIT values. The CIT and FO values were calculated as previously described [26, 27]. ΔFat oxidation was calculated by subtracting the fat oxidation value of pre-cold exposure from that of post-cold exposure.

### BAT measurements using IRT

Measurements were conducted during the winter and summer (December–March and June–September, respectively). The participants abstained from excessive drinking, staying up late, and exercising on the previous day. On the day of the experiment, the participants skipped breakfast and participated in the experiment in the morning (9:00–12:00). After a 30-min rest in a 27 °C room, the participants were asked to wear light clothing (thin disposable gowns) and rest in a sitting position for 90 min in a 19 °C air-conditioned room. Thermal images were acquired using a FLIR-E6-XT thermal imaging camera (Teledyne FLIR, Wilsonville, Oregon, USA). Infrared thermal images of the upper body were acquired at the beginning of and during cold exposure (10, 30, 60, and 90 min). The captured images were analyzed using FLIR tools (Teledyne FLIR). We used ∆Temp at 90 min of cold exposure as an indicator of BAT activity. ΔTemp was calculated by subtracting the skin surface temperature in the control chest region from that in the BAT-rich supraclavicular fossa region [28].

### SNP genotyping

In the FDG-PET/CT population, genomic DNA was prepared from buccal swab cells using the QIAamp DNA Mini kit (QIAGEN, Hilden, Germany), followed by ethanol precipitation using a Dr. GenTLE precipitation carrier (TAKARA BIO, Kusatsu, Japan). Genomic DNA was obtained from the saliva of the IRT population using the OraGene DNA kit (DNA Genotek, Ontario, Canada) and QIAamp DNA Midi kit (QIAGEN, Hilden, Germany).

SNP genotyping was performed using TaqMan SNP Genotyping Assays (Thermo Fisher Scientific K.K., Tokyo, Japan), a Light Cycler Probe Master, and a Light Cycler 96 instrument (Roche Diagnostics K.K., Tokyo, Japan). Eleven candidate SNPs of six gene regions—rs1137100, rs1892535, rs1022981, rs12405556, and rs4655518 (*LEPR*); rs2278426 (*ANGPTL8*); rs11573162 (*PLA2G2A*); rs6496589 (*PLIN1*); rs2298080 (*TBX15-WARS2*) and rs3790553 (*WARS2*); and rs174547 (*FADS1*)—were selected based on the literature (Table S4) [11, 12, 14–18]; these were reported as being under positive natural selection in high-latitude populations or populations living in cold climates. *LEPR* SNPs have strong linkage disequilibrium [29, 30]. We analyzed all the SNPs because they were inconclusive which were truly important.

### Statistical analysis

Agreement with the Hardy–Weinberg equilibrium was assessed using the *χ*^2^ goodness-of-fit test, and the significance level was set at 0.0045, considering the number of SNPs tested. Associations between SNP genotypes and BAT activity were evaluated using logistic or multiple linear regression models adjusted for age, sex, test month, or test season, interactions of age and sex, and ethnicity as appropriate, following a previous study [9]. In the FDG-PET/CT population, three genetic models were tested for each SNP, additive, dominant, and recessive, for the putatively selected allele (Table S4). Dependent variables without a normal distribution were log-transformed. *P*-values of less than 0.05 were considered to indicate statistical significance in the analyses of CIT, FO, and the IRT validation study. All statistical tests were two-sided. SPSS software (IBM, Tokyo, Japan) was used for all statistical analyses.

## Results

**Fig. 1:**
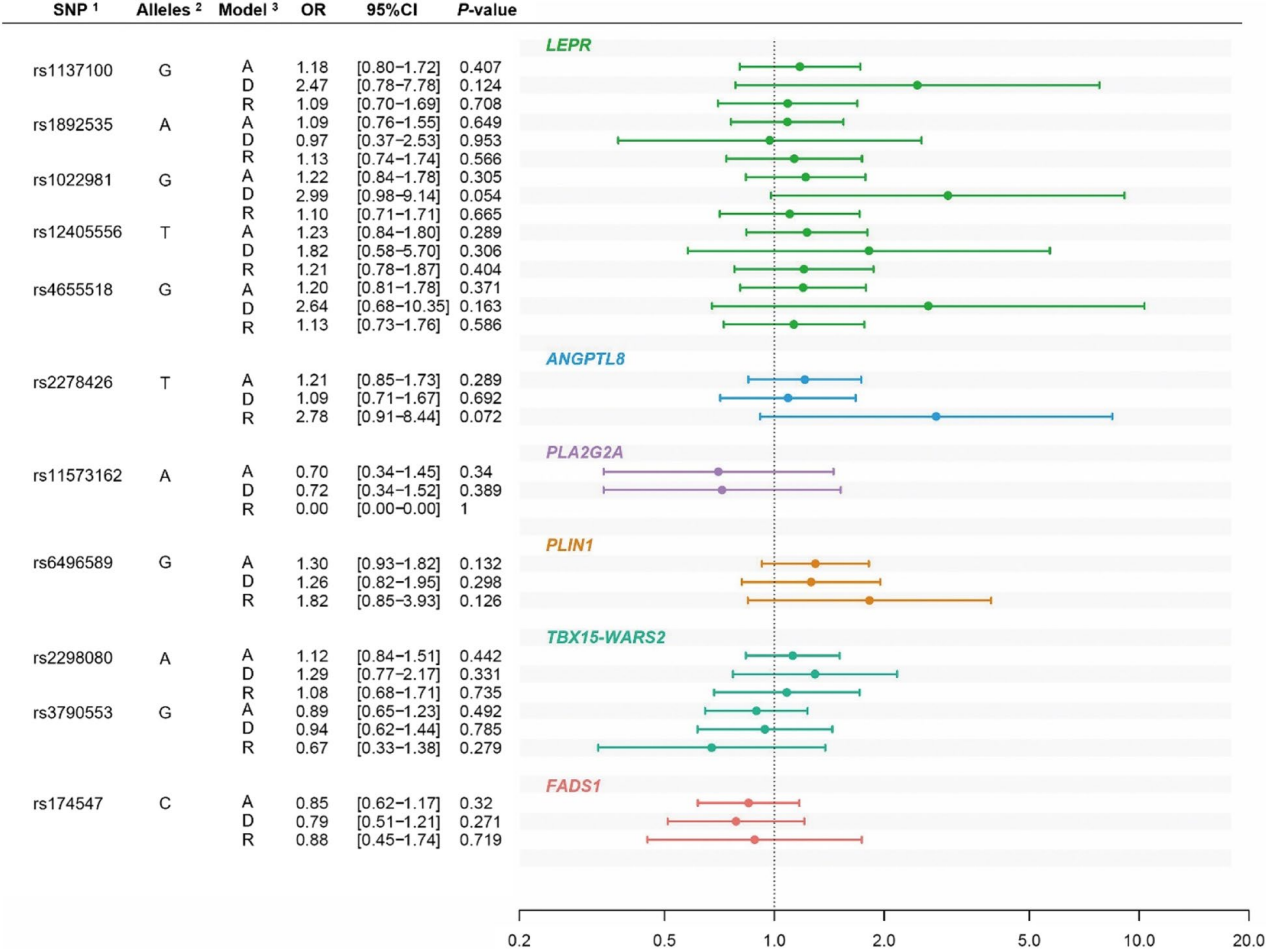
Results of the logistic regression analysis of BAT-positive/negative and SNP genotypes. The forest plot shows the association between genotype groups and BAT prevalence in the FDG-PET/CT population (*n* = 399). Binary variables representing BAT-negative or BAT-positive statuses were coded as 0 or 1, respectively. Sex (0 = female, 1 = male), the interaction term between age and sex (female: 0 × age, male: 1 × age), a dummy variable representing the study month (0 = December and March, 1 = January and February), and the genotype were included as independent variables. To consider multiple tests of the number of SNPs and genetic models, the significance level for the genotypes was set at *P* = 0.0015. ^1^Identifiers in the Single Nucleotide Polymorphism Database at the National Center for Biotechnology Information are shown. ^2^Putatively selected alleles of each SNP are indicated. ^3^The tested genetic models were as follows: A, additive model; D, dominant model; and R, recessive model for the putatively selected allele. SNP, single nucleotide polymorphism; OR, odds ratio; 95% CI, 95% confidence interval. Black dots and horizontal bars represent ORs in each model and their 95% confidence intervals (CIs), respectively

**Fig. 2:**
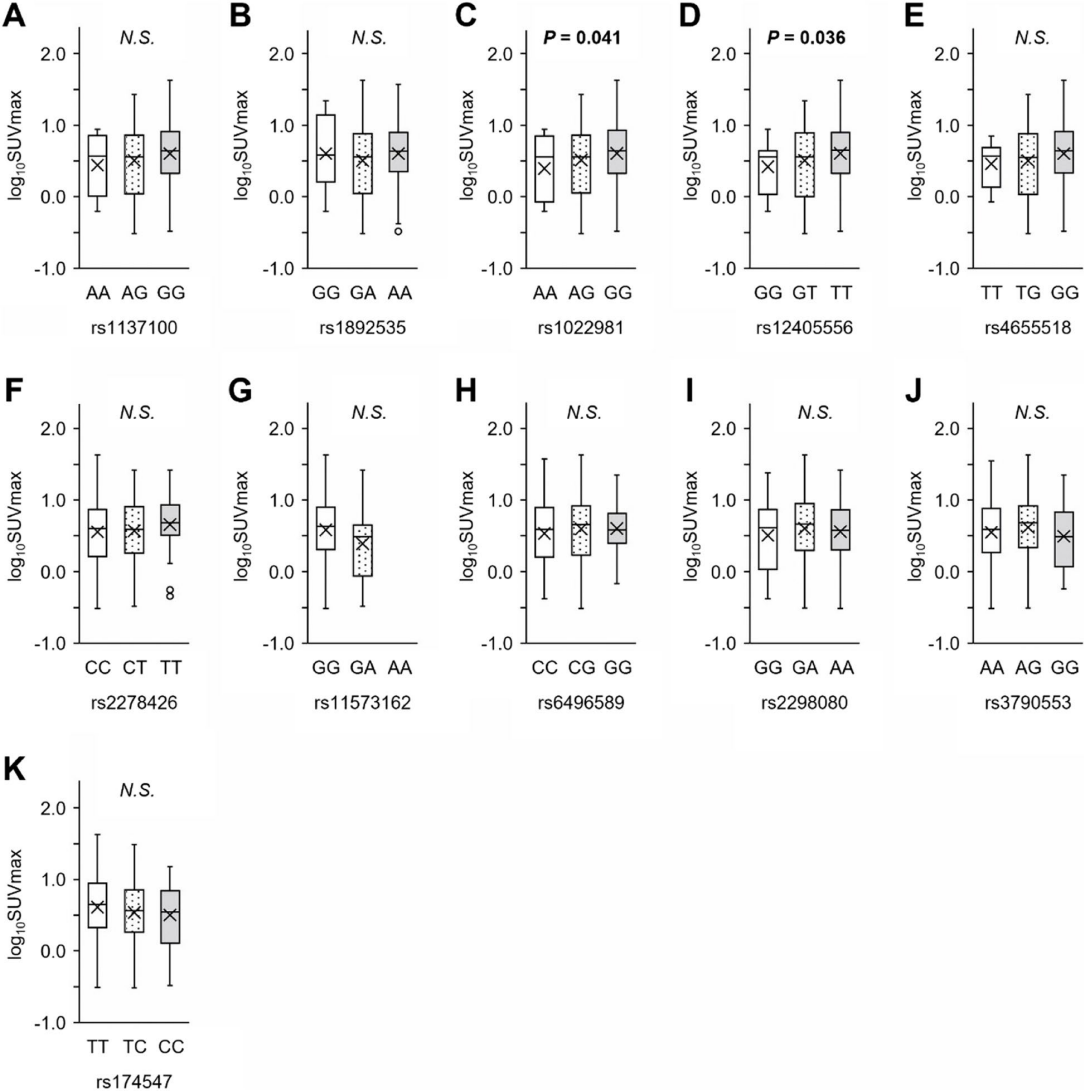
Results of the association study between SUV_max_ and SNP genotypes. **A**–**E** SNPs of *LEPR*; **F** SNP of *ANGPTL8*; **G** SNP of *PLA2G2A*. Note the absence of AA homozygotes in this population due to low minor allele frequency. **H** SNP of *PLIN1*; **I**, **J** SNPs of *TBX15* and *WARS2*; and **K** SNP of *FADS1*. Box plots show the distribution of log_10_SUV_max_ in the genotype groups of the FDG-PET/CT population (*n* = 325). In each box plot, genotypes are shown in the following order: homozygotes of the putatively selected allele (right), heterozygotes (middle), and homozygotes of the counter allele (left). Sex (0 = female, 1 = male), the interaction term between age and sex (female: 0 × age, male: 1 × age), a dummy variable representing the study month (0 = December and March, 1 = January and February), and the genotype were included as independent variables. Box plots show median values (central line), mean values (cross mark), and 75th and 25th percentiles (upper and lower boundaries, respectively). The largest and smallest values are represented as whiskers drawn from the box ends. Outliers are indicated by dots. *P*-values of genotypes in multiple linear regression models are shown. N.S., not significant

**Fig. 3:**
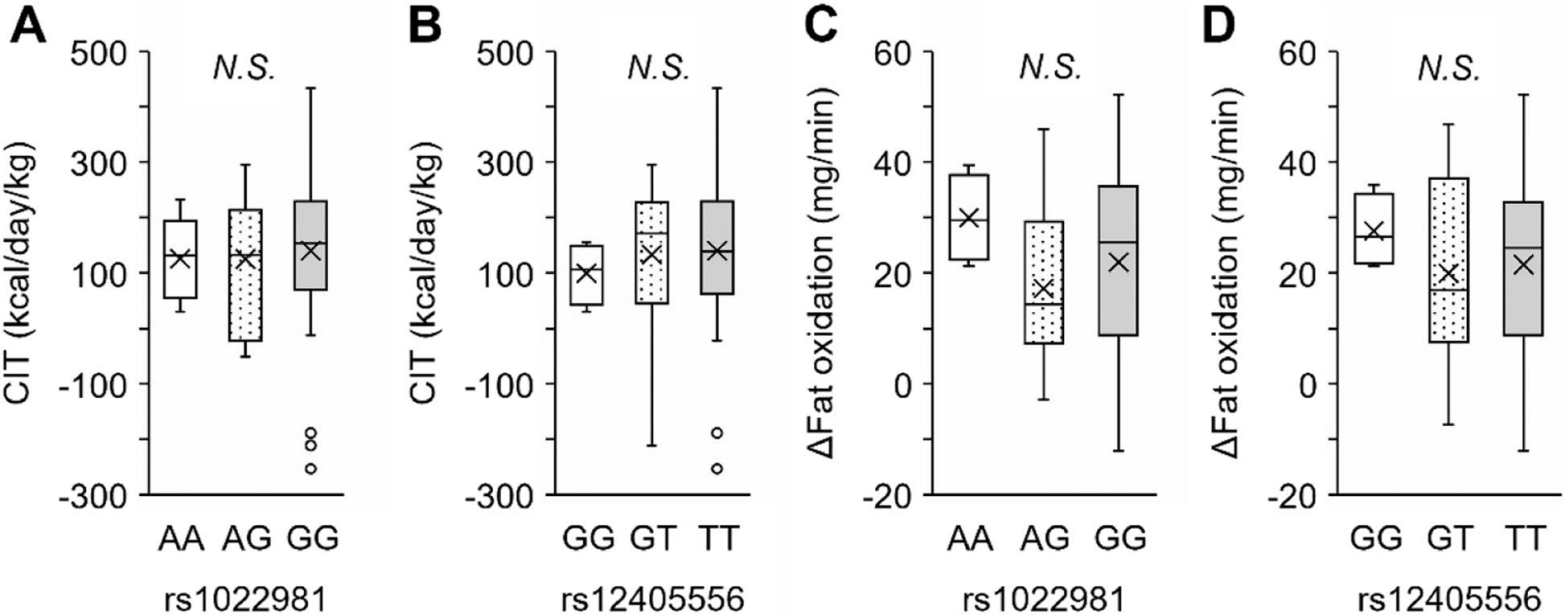
Results of the association study between genotypes and CIT and FO. The box plots show the distribution of values for capacity for CIT (kcal/day/kg) (**A**, **B**) and ∆Fat oxidation (mg/min) (**C**, **D**) in the genotype groups. Associations are shown for rs1022981 (*LEPR*) (**A**, **C**) and rs12405556 (*LEPR*) (**B**, **D**). In each box plot, genotypes are shown in the following order: homozygotes of the putatively selected alleles (right), heterozygotes (middle), and homozygotes of the counter allele (left). The box plot shows the median values (central line), mean values (cross marks), and 75th and 25th percentiles (upper and lower boundaries, respectively). The largest and smallest values are represented as whiskers drawn from the box ends. Outliers are indicated by dots. CIT, cold-induced thermogenesis; FO, fat oxidation. ΔFat oxidation was calculated by subtracting the fat oxidation value of pre-cold exposure from that of post-cold exposure. *P*-values of genotypes in multiple linear regression models are shown. N.S., not significant

**Fig. 4:**
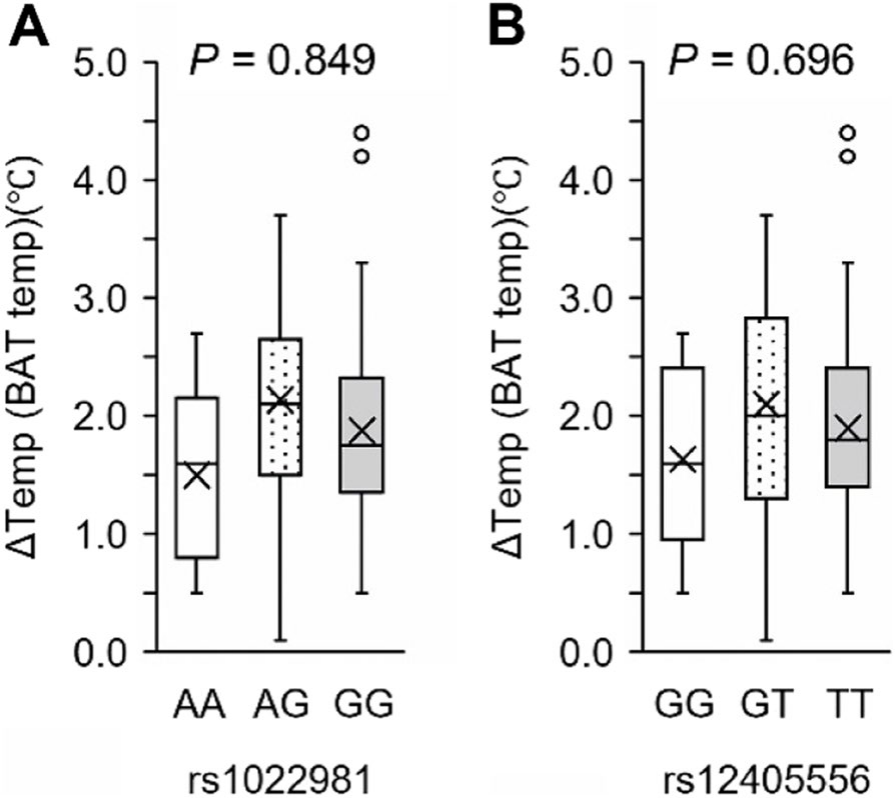
Results of the association study between BAT activity and rs1022981 (**A**) and rs12405556 (**B**) genotypes. The box plot shows the distribution of the ΔTemp (BAT temperature) values in the genotype groups at the end of the experiment. Age, sex (0 = female, 1 = male), season (summer = 0, winter = 1), ethnicity (Japanese = 0, other = 1), and genotype were included as independent variables. In each box plot, genotypes are shown in the following order: homozygotes of the putatively selected allele (right), heterozygotes (middle), and homozygotes of the counter allele (left). The box plot shows the median values (central line), mean values (cross marks), and 75th and 25th percentiles (upper and lower boundaries, respectively). The largest and smallest values are represented as whiskers drawn from the box ends. Outliers are indicated by dots. ΔTemp was calculated by subtracting the skin surface temperature in the control chest region from that of the supraclavicular fossa region. *P*-values of genotypes in multiple linear regression models are shown

Association analyses were performed on data from two independent populations, collectively comprising 483 individuals in whom BAT activity was measured using FDG-PET/CT (*n* = 399) or IRT (*n* = 84). The characteristics of the participants in each population are summarized in Tables 1, 2, S1, S2, S3, and S5. For the association test, we selected factors other than SNPs involved in BAT activity. In the FDG-PET/CT population, the BAT-positive group was younger than the BAT-negative group (25.6 ± 6.3 vs 29.6 ± 9.0, Mann–Whitney *U* test, *P* < 0.001, Table 1). It comprised a higher percentage of men (91.1% vs 77.6%, respectively, *P* < 0.001, Table 1). In the IRT population, women had a higher ∆Temp than men (Student’s *t*-test, *P* < 0.001; Table 2). Age was higher in the Japanese population than that for the other population (Mann–Whitney *U* test, *P* = 0.014, Table S3). Furthermore, body characteristics differed between sexes (Tables S1, S2, and S5). We performed association tests, including age and sex, with clear causal relationships with BAT activity as covariates. Based on the literature, we selected 11 SNPs from six genomic regions: *LEPR*, *ANGPTL8*, *PLA2G2A*, *PLIN1*, *TBX15-WARS2*, and *FADS1* (Table S4). These SNPs have been reported to be under positive selection in indigenous circumpolar populations, and the annotated genes are involved in BAT function in cellular and animal studies [11, 12, 14, 15, 17, 18, 21, 22, 31–33]. There was no deviation from Hardy–Weinberg equilibrium in the genotype frequencies of any SNPs (*P* > 0.05, Tables S4 and S6). First, we analyzed whether these SNPs affected BAT activity in the FDG-PET/CT population. No significant association was observed between genotype and BAT positivity (*P* > 0.1, Fig. 1). Considering that binarization might reduce the statistical power to detect the effect of SNPs on a quantitative trait, we tested the associations between these SNPs and the log-transformed SUV_max_ in 325 participants, excluding 74 for whom raw SUV_max_ data had not been recorded. Of the 11 SNPs, differences in the additive models were noted for two intronic SNPs of *LEPR*, rs1022981 and rs12405556 (*P* = 0.041, *β* =  − 0.108 and *P* = 0.036, *β* =  − 0.11, respectively; Fig. 2 and Table S7), although the association did not survive multiple testing correction. Carriers of the putative adaptive T allele at rs12405556, which showed a trend toward a higher SUV_max_, had significantly lower body fat percentage (BFP), plasma triglyceride levels, insulin levels, and HOMA-IR levels (Table 3). BAT metabolic activity uses lipids as substrates, and CIT and FO are auxiliary indicators of BAT activity [26, 27]. We performed association analyses using an additive model for CIT and FO in 56 male participants for two SNPs with different SUV_max_ values between the alleles. We failed to find any association between the two *LEPR* SNPs and CIT or FO (Fig. 3). Further association analyses were performed using data from an independent IRT cohort. There were no differences in BAT activity based on the presence of the allele (Fig. 4).

**Table 3:** Association of rs1022981 and rs12405556 (*LEPR*) and biochemical and anthropometric parameters in the FDG-PET/CT population

	All (*n* = 325)	**rs1022981**			*P*-value	**rs12405556**			*P*-value
		AA (*n* = 11)	AG (*n* = 111)	GG (*n* = 203)		GG (*n* = 11)	GT (*n* = 111)	TT (*n* = 203)	
Age (years)	26.0 ± 6.9	22.8 ± 3.6	26.8 ± 7.6	25.9 ± 6.7	0.126	22.9 ± 3.8	26.7 ± 7.7	25.9 ± 6.6	0.201
Height (cm)	171.3 ± 6.8	171.1 ± 7.7	171.7 ± 6.9	171.1 ± 6.7	0.638	170.9 ± 8.8	171.7 ± 6.7	171.1 ± 6.7	0.535
Weight (kg)	64.0 ± 10.1	63.5 ± 9.2	64.9 ± 11.1	63.6 ± 9.5	0.471	65.5 ± 10.6	64.8 ± 11.0	63.6 ± 9.5	0.217
BMI (kg/m^2^)	21.7 ± 2.8	21.7 ± 2.6	21.9 ± 2.9	21.7 ± 2.7	0.576	22.4 ± 2.6	21.9 ± 3.0	21.7 ± 2.7	0.290
BFP (%)	17.9 ± 5.6	18.9 ± 5.8	18.4 ± 5.8	17.5 ± 5.4	0.086	19.1 ± 5.4	18.6 ± 5.8	17.4 ± 5.5	0.034
T-Cho (mg/dl)	182.0 ± 29.3	187.3 ± 29.8	183.7 ± 32.5	179.9 ± 30.1	0.312	192.5 ± 26.7	184.6 ± 32.5	179.2 ± 30.1	0.175
HDL (mg/dl)	63.1 ± 12.3	59.0 ± 11.1	62.0 ± 11.7	63.7 ± 12.7	0.144	57.7 ± 8.5	62.4 ± 11.9	63.6 ± 12.7	0.142
TG (mg/dl)	74.2 ± 38.5	95.6 ± 64.7	77.0 ± 40.7	72.3 ± 37.0	0.081	112.0 ± 76.2	76.9 ± 37.7	71.5 ± 36.8	0.006
Glucose (mg/dl)	82.4 ± 6.4	81.3 ± 6.6	82.5 ± 6.0	82.6 ± 6.6	0.668	81.3 ± 6.3	82.4 ± 5.9	82.7 ± 6.7	0.504
HbA1c(NGSP) (%)	5.1 ± 0.3	5.0 ± 0.4	5.1 ± 0.2	5.1 ± 0.3	0.519	5.1 ± 0.4	5.1 ± 0.2	5.1 ± 0.3	0.803
Insulin (µIU/ml)	4.6 ± 2.7	5.5 ± 2.5	4.8 ± 2.8	4.4 ± 2.6	0.150	5.7 ± 2.7	5.0 ± 2.9	4.3 ± 2.5	0.024
HOMA-IR	0.9 ± 0.6	1.1 ± 0.6	1.0 ± 0.6	0.9 ± 0.6	0.179	1.2 ± 0.6	1.0 ± 0.6	0.9 ± 0.6	0.035

The analysis included data from 325 participants in the FDG-PET/CT population. Continuous data are expressed as the mean ± standard deviation. HOMA-IR was calculated from fasting glucose × fasting insulin/405. *P*-values were calculated from statistical tests as follows: age, Kruskal–Wallis test; other traits, multiple linear regression models adjusted for sex and age (non-normally distributed trait values were log-transformed before the tests)

*BMI* Body mass index, *BFP* Body fat percentage, *T-Cho* Total cholesterol, *HDL* High-density lipoprotein, *TG* Triglycerides

## Discussion

Of the six loci tested, *LEPR* showed a tendency for carriers of adaptive alleles to have higher BAT activity in the FDG-PET/CT test (Fig. 2 and Table S7). Notably, the slight associations with BFP, plasma triglyceride, insulin, and HOMA-IR levels (Table 3) were consistent with the negative correlation between BAT activity and body fat [23, 24]. Leptin is produced in adipose tissues and has pleiotropic functions such as controlling appetite and regulating energy balance via interactions with the sympathetic nervous system [34–36]. It has also been reported to mediate sympathetic activation of BAT [34] or the induction of beige adipocytes [37]. The lower BFP observed in carriers of the putative adaptive alleles contrasts with a report that high BFP is associated with increased levels of insulation [38], indicating a requirement for thermogenesis in cold environments. The healthier body composition and blood biochemical parameters observed in carriers of this allele could be attributed to enhanced energy expenditure by leptin-mediated BAT activation [34, 37].

Both *LEPR* SNPs have been identified as quantitative expression trait loci (eQTL) in peripheral blood cells [39]. Rs12405556 is in a putative enhancer of *LEPR* in the liver, and rs1022981 has strong linkage disequilibrium with putative promoter and enhancer SNPs of *LEPR* in some tissues [30]. Other SNPs in strong linkage disequilibrium with these positively associated SNPs could be genuinely effective SNPs. Moreover, increased transcript levels of adaptive alleles have been observed in several tissues [40]. Thus, it is a potential contributor to the maintenance of deep body temperature by promoting *LEPR* transcript expression. In contrast, *LEPR* alleles were not associated with significant differences in CIT, FO, or BAT activity as assessed using IRT. These apparently conflicting results may be explained, in part, by the limited number of participants, which likely restricts the statistical power. The thermal images acquired by IRT may be affected by focal fat thickness and blood flow; thus, these factors need to be controlled in future studies. The BFP differed between the sexes in terms of insulation properties. Given these findings, *LEPR* may be involved in cold adaptation through BAT activity. However, further studies with larger sample sizes are required.

Another notable finding of the present study was the locus involving *TBX15* and *WARS2*, which has been associated with a wide range of traits and is thought to be involved in cold adaptation in the Greenland Inuit [15]. The putative adaptive allele at this locus was likely introgressed from Denisovan hominins, who were already distributed in high latitudes in East Eurasia before the arrival of modern humans [14, 15]. *Tbx15* and *Wars2* participate in the differentiation of brown adipocytes in mice [21, 41]; however, no association was observed between the capacity for BAT thermogenesis and these SNPs in the present study. This locus was previously found to be significantly associated with facial and body morphology, such as lip thickness, height, and waist-to-hip ratio [32, 42]. Facial morphology appears to play an important role in cold adaptation in humans [43, 44], and a previous study has suggested that body temperature is regulated by insulation when the BFP is high [38]. Thus, natural selection at this locus in the Greenland Inuit may target phenotypes related to insulation, such as body fat distribution and facial morphology.

*ANGPTL8* plays a role in the production of very low-density lipoproteins by regulating lipolysis and lipid accumulation in the liver [17, 45]. *PLIN1* is expressed in the adipocytes and plays essential roles in lipolysis and lipogenesis [46]. The positive selection in these genes may predate human dispersal into subarctic regions. Furthermore, the putative adaptive alleles were detected at a low frequency in Africa, suggesting that variations arose long before selection began. Thus, the involvement of ecological factors other than low temperature is a reasonable consideration [12, 17]. *PLA2G2A* participates in the generation of free fatty acids by hydrolyzing phospholipids and influences circulating lipid levels. *FADS1* is involved in fatty acid metabolism modulation. This gene is likely linked to adaptation to animal-rich diets, which are tied to the encoding of delta-5 desaturase enzymes that increase unsaturation of polyunsaturated fatty acids more efficiently, as evidenced by observations in the Inuit, who relied on diets based on nutrients sourced from marine mammals [14]. Based on these reports, aspects beyond adaptation to cold conditions, such as adaptation to unique diets and efficient energy storage in scenarios where food is not readily accessible, may explain the natural selection of these genes.

It should be clarified whether the loci tested here interact with other factors that influence BAT activity. Previous studies have reported sex-based differences in BAT activity and the CIT [47, 48]. Sex also has a substantial impact on body fat deposition, which can be influenced by the energy-dissipating nature of BAT and possibly affects BAT activity by altering the insulation properties of the body [49]. In particular, *LEPR* may need to be examined in detail since LEPR participates in the regulation of food intake, energy expenditure, and the function of gonadal glands, all of which showed sex differences [50]. The sex-specific effects of variants at the *LEPR* on the development of obesity have been reported [51]. Although our association analysis was adjusted for sex, further association analyses with larger sample sizes are required to address these issues.

This study had some limitations. This study was based exclusively on SNPs documented in previous population genetics studies. The SNPs tested may not have adequately captured the variant that was the actual target of natural positive selection. Moreover, how variations in BAT activity are inherited in modern humans remains unclear. If this trait is highly polygenic, our analysis may have limited statistical power because of the relatively small sample size. In particular, the CIT and FO measurements and association analyses were conducted only in 56 males. Further studies of data from larger populations are required. Increasing the sample size is indispensable for assessing the interactions between SNPs and factors of interest, such as sex, tested season, and ethnicity. Differences in dietary behavior may affect BAT activity [52]; however, dietary surveys were not conducted in this study. The inclusion of dietary information is essential for clarifying the effects of *LEPR* SNPs on BAT activity. Finally, variants specific to circumpolar populations could not be analyzed.

## Conclusions

The present study demonstrates that *LEPR* may play a role in cold adaptation. In contrast, we did not find robust evidence for the involvement of other previously identified “cold-adaptive” genes, including *TBX15*, in the variability of thermogenic phenotypes. If there are traces of natural selection in high-latitude and low-temperature regions and the genomic regions are involved in BAT differentiation and function, it does not necessarily mean that they are directly involved in the diversity of human thermogenesis. We substantiated the importance of verifying the results of genomic analyses using actual human physiological information. This study deepens our understanding of the genomic basis of cold adaptation in humans and provides intriguing insights into human adaptation and the evolution of thermogenetic diversity.

### Supplementary Information

Additional file 1: Table S1. Sex differences of participants in the FDG-PET/CT population. Table S2. Sex differences of participants in the IRT population. Table S3. Nations differences among participants in the IRT population. Table S4. SNP genotyping results in the FDG-PET/CT population (*n* = 399). Table S5. Sex differences of biochemical parameters in the FDG-PET/CT population (*n* = 325). Table S6. SNP genotyping results for rs1022981 and rs12405556 (*LEPR*) in the IRT population (*n* = 84). Table S7. Results of the association study in the FDG-PET/CT population (*n* = 325). Fig. S1. Summary of the tested *LEPR* SNPs.

## Data Availability

The data analyzed during the current study are not publicly available owing to ethical regulations but will be available from the corresponding author upon reasonable request.

## Abbreviations

95% CI: 95% Confidence interval
*ANGPTL8*: Angiopoietin-like-8 gene
BAT: Brown adipose tissues
BFP: Body fat percentage
BMI: Body mass index
CIT: Cold-induced thermogenesis
EAs: East Asians
eQTL: Quantitative expression trait loci
*FADS1*: Fatty acid desaturase 1 gene
FDG-PET/CT: Fluorodeoxyglucose-positron emission tomography and computed tomography
FO: Fat oxidation
HDL: High-density lipoprotein
IRT: Infrared thermography
MAF: Minor allele frequency
Models: A, Additive; D, dominant; R, recessive
N.S.: Not significant
OR: Odds ratio
*PLA2G2A*: Phospholipase A2 group 2A gene
*PLIN1*: Perilipin1 gene
SNP: Single nucleotide polymorphism
SUV_max_: Maximum standardized uptake value
*TBX15*: T-box transcription factor 15 gene
T-Cho: Total cholesterol
TG: Triglyceride level
*UCP1*: Uncoupling protein 1 gene
*WARS2*: Tryptophanyl tRNA synthetase 2 gene
